# Metabarcoding profiling of microbial diversity associated with trout fish farming

**DOI:** 10.1038/s41598-020-80236-x

**Published:** 2021-01-11

**Authors:** Mohamed A. A. Mahmoud, Mahmoud Magdy

**Affiliations:** 1 Chair of Aroma and Smell Research, Department of Chemistry and Pharmacy, Emil Fischer Center, Friedrich-Alexander-Universität Erlangen-Nürnberg, Henkestraße 9, 91054 Erlangen, Germany; 2 Sensory Analytics Department, Fraunhofer Institute for Process Engineering and Packaging IVV, Giggenhauser Str. 35, 85354 Freising, Germany; 3 Agricultural Biochemistry Department, Faculty of Agriculture, Ain Shams University, 68 Hadayek Shobra, Cairo, 11241 Egypt; 4 Genetics Department, Faculty of Agriculture, Ain Shams University, 68 Hadayek Shobra, Cairo, 11241 Egypt

**Keywords:** Water microbiology, Next-generation sequencing, Freshwater ecology, Microbial ecology

## Abstract

Earthy and musty off-flavors are routinely observed in farmed trout worldwide. The microbial association to the production of those off-flavors was previously reported. The current manuscript aimed to catalog the microbial enrichment (eukaryotes and prokaryotes) in semi-intensive aquaculture freshwater sources that might influence the trout aquaculture quality production. The 16S rRNA and ITS metabarcoding analyses were applied on the inflow- and pond-water samples from trout farms previously recorded a malodor fish products and located alongside Moosach and Sempt Rivers in Bavaria province, Germany. The results showed that more than 99% of the detected prokaryotic OTUs (Operational Taxonomic Unit identification) were bacteria as of ~ 75.57% were Proteobacteria, and ~ 14.4% were Bacteroidetes. Meanwhile, 118 out of 233 of the eukaryotic OTUs were known species. Of these, ~ 45% were plant pathogens, and ~ 28% were mushroom/yeasts. Based on the comparative analysis between inflow- and pond-water samples, several pro- and eukaryotic microorganisms that affect the trout aquaculture water quality and industry have been detected, including the malodor-producing microorganisms, e.g., Cyanobacteria and Actinobacteria, along with fish infectious microorganisms, e.g., *Chilodonella cyprinid*, *Metschnikowia bicuspidate*. Additionally, the effect of the human- and industrial-related activities around the sampling area on the microbiota of the investigated farms were highlighted.

## Introduction

The high demand for fish meat has promoted rapid development in the supply chain to the point that about 47% of the world fish consumption originates from aquaculture farming^1^. The downside of this development is the increasing amounts of waste (dissolved organic and inorganic waste and particulate waste) originated by aquaculture practices and its related adverse impacts on our environment, i.e., disrupting the homeostatic microbial community of aquaculture water^2,3^. Although the dissolved waste can quickly disperse and dilute in the water column, the particulate organic waste sinks to the ground to form enriched sediments^4^. This leads to the accumulation of nutrients that stimulates microbial activity along with the sediments layer and leads to deoxygenation of water at the ground of ponds and lakes^4^. The combined effect of nutrient leaching and the depletion of dissolved oxygen leads to a significant change in the composition of the ecosystem living communities^4^.

Another negative consequence from the aquaculture industry point of view is the possible formation of malodors in aquaculture water due to the fluctuation of nutrients, which leads to the production of fish with poor sensorial qualities^5,6^. Furthermore, the resulting malodors can reside in water for an extended time due to their relative stability to chemical and/or biological degradation^7,8^. The above mentioned negative impacts could be cumulative; thus, present extended effects from the upstream to the downstream farms^3^. It is essential to highlight that this theory (the off-flavor problem rises in the upstream could affect fish quality in downstream farms) has not been sufficiently discussed in German freshwater aquaculture.

Biodiversity focuses on the richness and distribution of organisms within a specific range^9^. Any alteration to the environmental conditions within that range might disrupt any homeostatic microbial community; thus, changes in their richness and distribution because of fluctuations in species favored the new conditions (e.g., dietary changes induce transient fluctuations in gut microbiota^10^). Microbial ecology studies have undergone significant improvements due to, e.g., environmental DNA technologies, such as next-generation sequencing (NGS)^11–13^. This has led to a vast expansion of the metagenomics and metabarcoding that define microbial communities based on DNA analysis from an environmental sample without prior need for culturing^11^. For instance, metabarcoding has been used to develop a biotic index that helps monitor benthic organic enrichment in salmon farms located in separate bioregions^12^ and assess high and low UV dosage treatments on the microbial community in Pacific oyster hatcheries^13^. Furthermore, the advancements in statistical/computational software and databases with its ability to exploit a massive amount of data generated by next-generation sequencing have greatly improved species diversity delimitation^14^.

This manuscript is the fourth part of article series that focused on fish feed composition and their impact on water contents from odor and taste compounds and their accumulation in fish (meat and skin). Where numerous off-odors were identified for the first time in aquaculture water and commercial fish feed; and their accumulation in rainbow trout fish meat and skin have been confirmed using the state-of-the-art aroma analytics, 1- and 2-dimensional gas chromatography–mass spectrometry combined with sensory analysis^15–17^. Although these studies have confirmed fish feed as a potential off-flavor source in the aquaculture industry, they did not highlight the effect of the surrounding environment on the microbial community concerning the accumulation of these off-odors.

Fish malodor is routinely observed in several cultivated fish species^18^. This problem is frequently associated with the exogenous accumulation of the earthy and musty off-flavors (2-methylisoborneol and geosmin) from water in fish^19,20^. These compounds' primary producers are species belonging to Actinobacteria and Cyanobacteria phyla^6–8,18,19^. Recent reports indicated that other species might also contribute to fish malodor (e.g., members of the genera *Myxobacteria* and *Sorangium* of the phylum Proteobacteria;^21^). However, the contribution of these two genera in the malodor formation in German trout aquaculture are yet to be covered.

The present study focused on fish aquaculture in the German state of Bavaria, where most of the country freshwater fish are cultivated^22^. We aimed to profile both prokaryotic (16S) and eukaryotic (ITS) microbial community of three different semi-intensive freshwater trout farms (where fish malodors had been previously reported^15–17^ using the metabarcoding approach. In order to (1) identify potential microbes associated with the fish malodors formation, (2) detect the possible cumulative impacts of the surrounding environment on the water downstream of the aquaculture facility, and b) acquire extended information to hypothesize the role of fish feed on fish malodor formation in the trout aquaculture industry in Germany.

## Results

### General microbial profile

For the 16S libraries, the six samples recorded 1,054,909 reads, with a length between 51 to 533 bp and an average of 458. In general, the number of clustered sequences was 652,899 (61.89%), while the number of replicated reads was 140,196 (13.29%). The number of classified sequences was 1,047,271 (99.28%) while only 7,155 sequences exhibited ‘unassigned’ (0.68%). The quality control of classification, in this case, the alignment similarity, was between 75 and 100%, while the majority exceeded 80%. Based on the 16S rRNA dataset, prokaryotic OTU identification pipeline, > 99% of the detected OTUs belonged to the bacteria domain. A total of 1318 species belonging to 17 phyla were detected in all samples. The most abundant were Proteobacteria (75.57%), Bacteroidetes (14.40%), Actinobacteria (0.94%), Verrucomicrobia (0.62%), and Cyanobacteria (0.25%).

**Figure 1:**
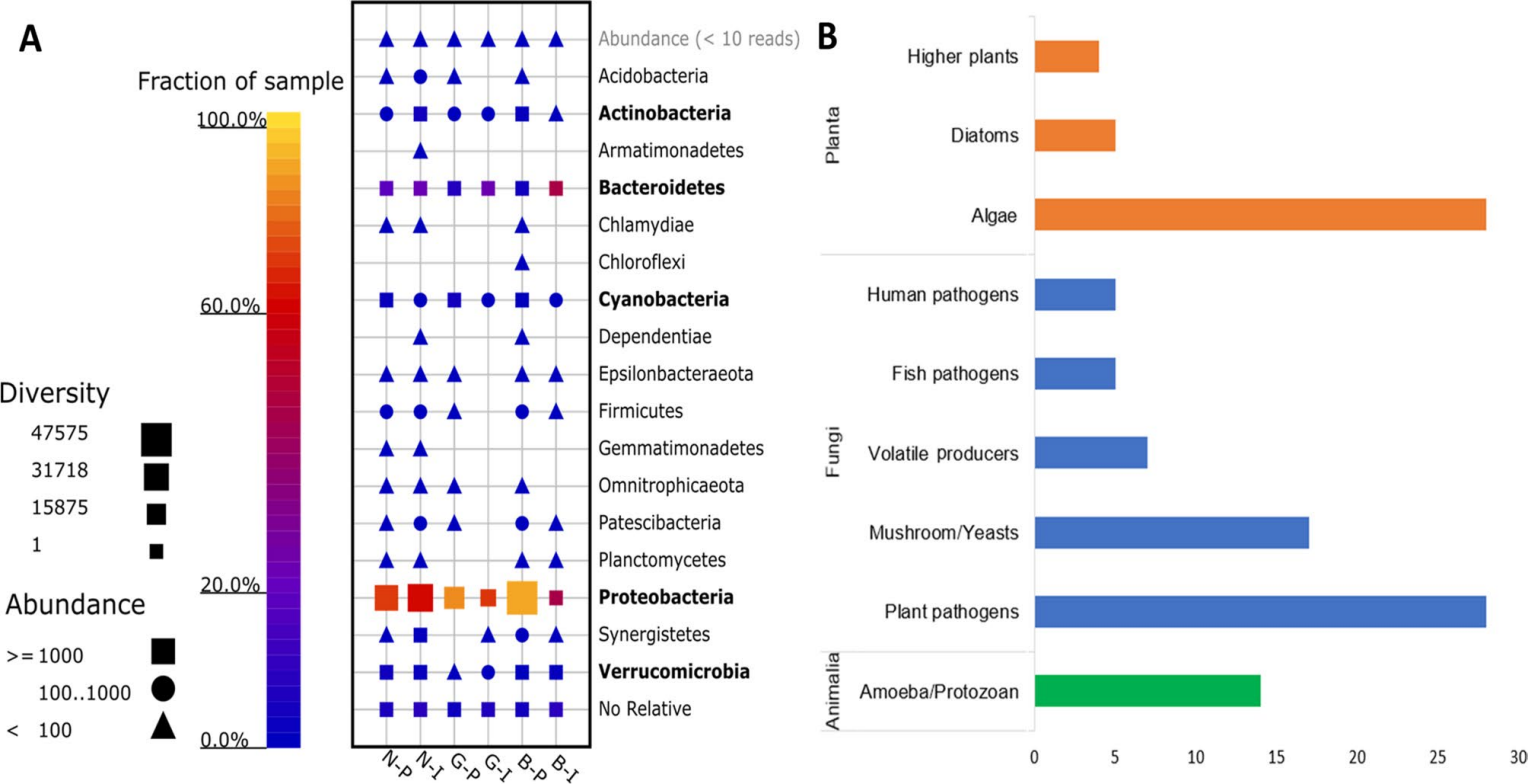
Microbial diversity detected by the metabarcoding analysis. The relative abundance of identified bacterial OTUs among the six water samples, where top abundant bacterial phyla are written in bold (**A**). The histogram plot shows the identified eukaryotic groups per domain (Planta, Fungi, or Animalia). The target group in the eukaryotic metabarcoding analysis was the fungal group distributed according to their prominent role and function (BBMerge–accurate paired shotgun read merging via overlap).

For the ITS2 libraries, the six samples recorded 2,193,552 reads, the assembled contigs length between 201 and 482 with an average of 292. In general, the number of generated consensus sequences ranged between seven and 47 per sample. In total, 191 (~ 75%) were successfully identified with pairwise identity ranging from 82 to 100%, while 63 sequences (~ 25%) hit an uncultured species (Supplementary Fig. 1). Based on the customized eukaryote OTU identification pipeline, 118 out of 233 were known species, ~ 55% of the identified OTUs belonged to the kingdom Fungi, ~ 33% belonged to the kingdom Plantae, and ~ 12% belonged to the kingdom Animalia. Due to the high diversity among the detected OTUs, fungi were grouped by their major function rather than their taxonomical position. The most represented Fungi group was the plant pathogens (~ 45%), followed by mushrooms/yeasts (~ 28%), volatile producers (~ 11%), fish pathogens (~ 8%), and human pathogens (~ eight%) of the total fungal OTUs (Fig. 1).

### Comparative metabarcoding analysis

#### Microbial diversity indices

**Figure 2:**
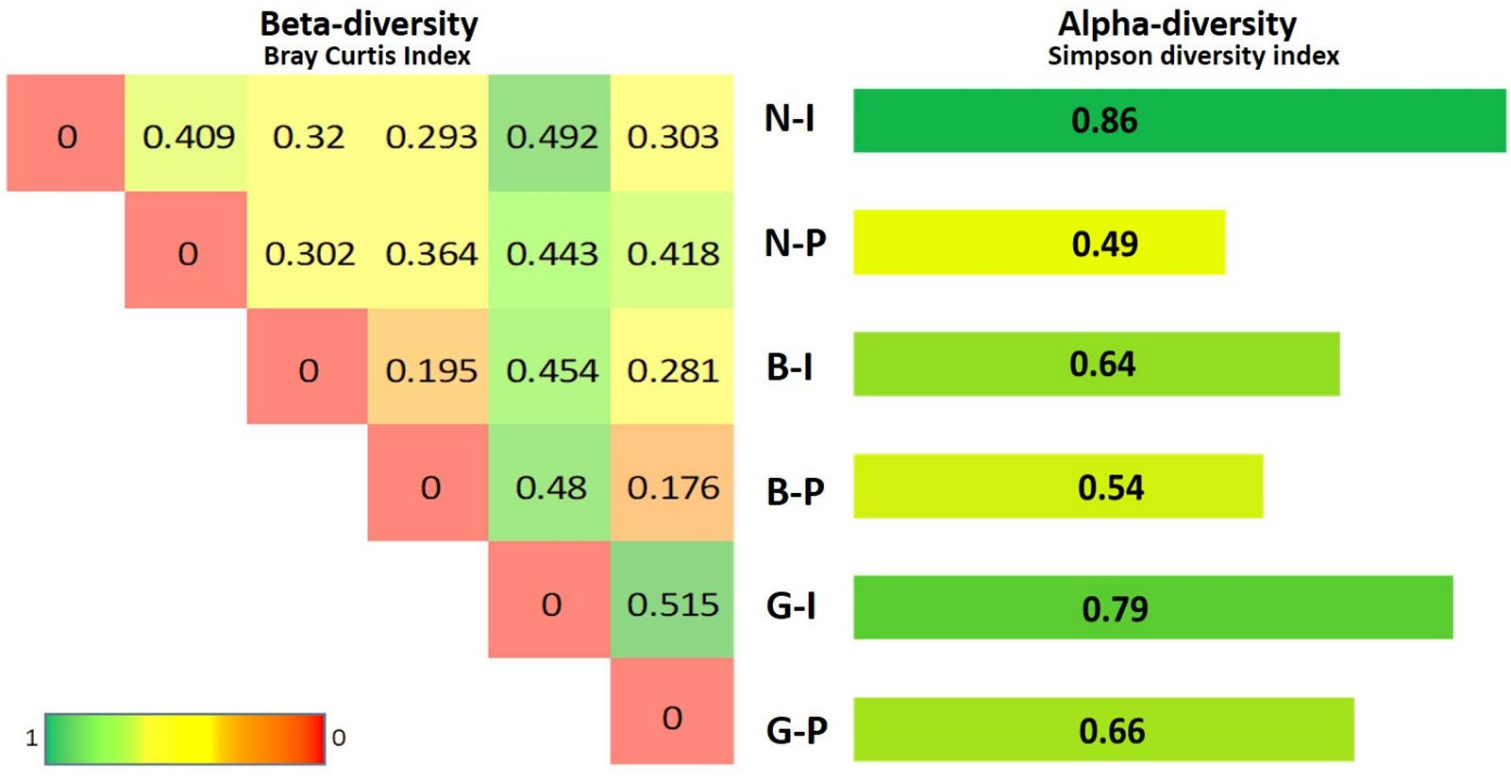
Alpha and beta-diversity of identified bacterial communities are estimated according to the Simpson diversity index and Bray Curtis, respectively. The three locations (N, G, and B) from two different sources, the inflow- (I) and pond-water (P), are shown.

For the 16S, the average alpha-diversity was estimated for each source; P-source showed a lower alpha-diversity than I-source. For 16S rRNA, the Simpson index values of the P-sources were lower than the I-sources (Fig. 2). Specifically, in samples from location N compared to the rest of the samples (0.86 for N-I and 0.49 for N-P). For B and G locations, D-index was 0.64 (B-I), 0.54 (B-P), 0.79 (G-I), and 0.66 (G-P). Based on sample locations, beta-diversity values of location G were the highest, while location B was the lowest. The G-I showed the highest beta-diversity for inter-location values, followed by N-P, N-I, G-P, B-I, and B-P (Fig. 2). This might indicate that the changes in a pond diversity are contributed by sources other than the inflow-water (e.g., transferred by juvenile fish or fingerlings, or the introduction of fish feeds).

#### Species occurrences and distributions

The identified species were detected in all locations (common) or exclusively detected in (a) either I-source or P-source samples, (b) exclusively found in one location regardless of the water source, (c) uniquely recorded in one sample.

A total of 1318 bacterial species were delimited. In which 1074 species were identified from I-sources, with the highest number was found in B-I (774), followed by N-I (669) and G-I (548). The number of detected species from P-sources was 1006 across the three locations. The highest number of species was found in B-P (882), followed by N-P (553) and G-P (415) locations. The highest number of species was 1081 from location B (321 were unique), followed by 804 species from location N (124 were unique) and 665 species from location G (94 were unique), regardless of the water source (i.e., species detected in one or both samples of each location). Locations N and B had 208 common species, while locations B and G shared 99 species, and locations N and G shared only 19 species. A total of 453 species were common among the three locations, of which 442 were common regardless of the water source.

**Figure 3:**
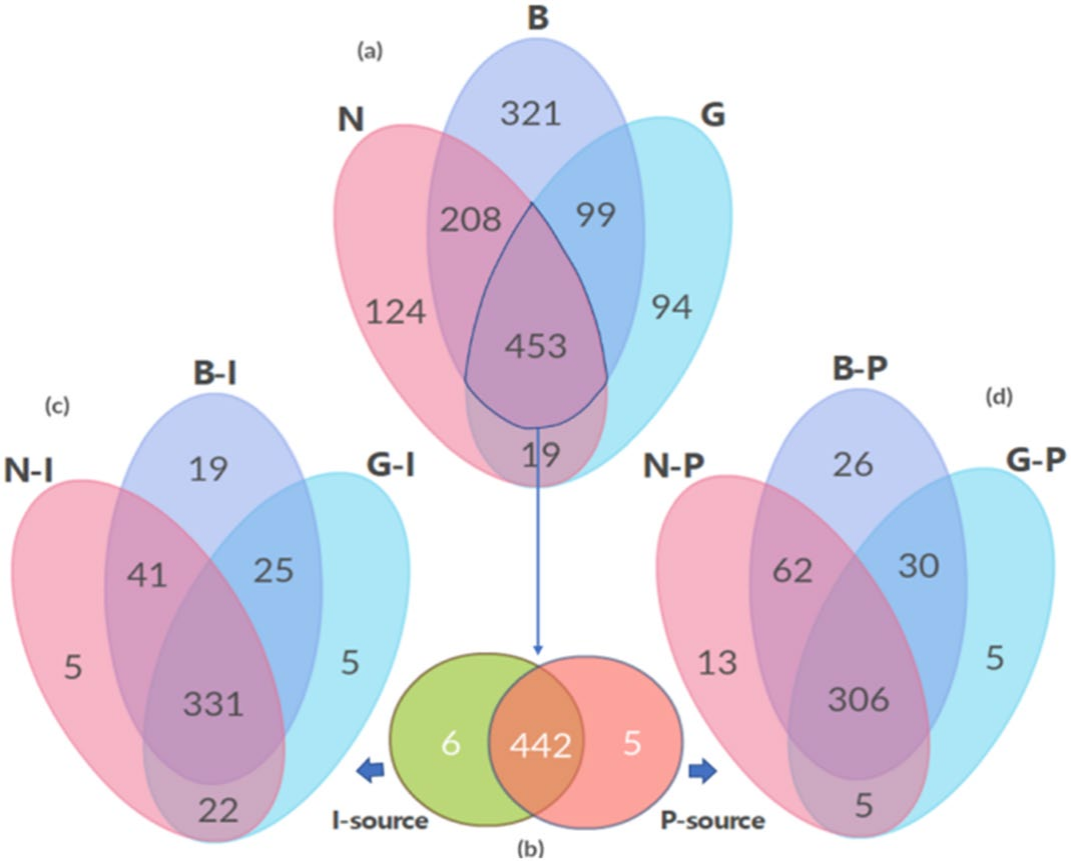
Venn diagram of shared and uniquely identified OTUs among the three sampling locations (a), where the common OTUs were counted by source (I or P; b). For each source, OTUs were separated by sample locations, respectively (c & d).

Out of the 453 common species, six species were exclusively detected from I-sources samples. Among the 442, the highest number of species was 415 from B-I (19 unique), followed by 399 from N-I (five unique) and 383 from G-I (five unique). Forty-one species were common between N-I and B-I, 25 between B-I and G-I, and 22 between N-I and G-I samples. Three hundred thirty-one species were common among samples N-I, B-I, and G-I. The number of the exclusively detected species in P-sources samples was five from the three locations. Among 442 species, the highest number of species was 424 from B-I (26 unique), followed by 386 from N-I (13 unique) and 346 from G-I (five unique). A total of 62 species were common between N-I and B-I, 30 between B-I and G-I, and five between N-I and G-I samples, while 306 were common among N-I, B-I, and G-I samples (Fig. 3).

Based on the fungal community, 233 OTUs were detected, 84 were unknown fungi (36%), 31 uncultured fungi with at least one taxonomical rank is known (14%), and 118 species were successfully identified (50%). The detected OTUs in I-sources was 123, with the highest number of OTUs from B-I (79), followed by N-I (37) and G-I (seven). The number of detected OTUs in P-source was 110 from the three locations. The highest number of OTUs was found in B-P (46), followed by N-P (42) and G-P (22) locations. Regardless of the water source, the highest number of OTUs was 125 from location B (24 were unique), followed by 79 OTUs from location N (30 were unique), and 29 OTUs from location G (11 were unique). Locations N and B shared two OTUs, while B and G shared one OTU, and N and G locations shared no OTU. Only one uncultured fungus was shared among the three locations. Between both water sources, based on known and uncultured fungi with at least one taxonomical rank, 20 species were common between the I- and P-sources, 56 species unique for I-sources, and 49 species unique for P-sources. However, none were commonly found among the P-source from the three sample source locations. Fungal OTUs number was following the bacterial OTUs number per sample, reflecting the homogenized overall diversity within each water sample.

### Microbial diversity unique to trout aquaculture water

Due to the lack of common eukaryotic OTUs among the P-source sites, the following analysis only focused on the prokaryotic species. The six exclusively identified bacterial species from the I-source belonged to three phyla, Proteobacteria, which has four species (Burkholderiaceae bacterium belong to MWH-UniP1 aquatic group, Caulobacteraceae bacterium, Hyphomonadaceae bacterium, and Rhodospirillales bacterium), one from phylum Bacteroidetes (Spirosomaceae bacterium) and one from phylum Firmicutes (*Solibacillus* sp.). For the P-source samples, the five exclusively species among the three locations belonged to two phyla, Bacteroidetes (*Ekhidna* sp., *Polaribacter* sp., and Sphingobacteriaceae bacterium) and Proteobacteria (*Thalassotalea* sp. and *Paraherbaspirillum* sp.).

Among the commonly-shared species, the one-tail distribution student t-test was applied to identify significantly different bacterial species between the two water sources (Table 1). A total of 15 species belonged to two phyla, and 12 families were significantly different between the I- and P-source samples. The phylum Bacteroides (significant at average; *p* value of 0.001) included eight species: *Marinoscillum* sp. (Cyclobacteriaceae), *Dysgonomonas* sp. (Dysgonomonadaceae), *Paludibacter* sp. (Paludibacteraceae), Saprospiraceae bacterium, *Mucilaginibacter* and *Pedobacter* (Sphingobacteriaceae), *Lacihabitans* (Spirosomaceae), uncultured ST-12K33 (unknown family), and *Empedobacter* (Weeksellaceae); all of the aforementioned species were less represented in I-source and more represented in P-sources. In the case of the other phylum (Proteobacteria), eight species were found to be significant. Three species: *Simplicispira* sp. (Burkholderiaceae), *Amaricoccus,* and *Thioclava* (Rhodobacteraceae) were up-represented in I-source and while four species: *Alicycliphilus* and *Caenimonas* (Burkholderiaceae), *Orientia* sp. (Rickettsiaceae), and Sphingomonadaceae bacterium were up-represent in P-source.

**Table 1:** Significantly differentiated bacteria (*p* > 0.05) as determined via a t-test, ordered by classification.

Bacteria classification*	Relative abundance (%; mean ± SEM)						*p* value**
	Overall	± SEM	I-source	± SEM	P-source	± SEM	
**Bacteroidetes**	**0.477**	**0.365**	**0.159**	**0.117**	**0.796**	**0.124**	**0.001**
**Cyclobacteriaceae**	**0.001**	**0.001**	**0.001**	**0.001**	**0.002**	**0.000**	**0.015**
Marinoscillum	0.001	0.001	0.001	0.001	0.002	0.000	0.015
**Dysgonomonadaceae**	**0.001**	**0.001**	**0.000**	**0.000**	**0.001**	**0.000**	**0.046**
Dysgonomonas	0.001	0.001	0.000	0.000	0.001	0.000	0.046
**Paludibacteraceae**	**0.035**	**0.024**	**0.015**	**0.006**	**0.056**	**0.015**	**0.013**
Paludibacter	0.035	0.024	0.015	0.006	0.056	0.015	0.013
**Saprospiraceae**	**0.227**	**0.183**	**0.071**	**0.055**	**0.384**	**0.084**	**0.004**
Uncultured	0.227	0.183	0.071	0.055	0.384	0.084	0.004
**Sphingobacteriaceae**	**0.176**	**0.141**	**0.056**	**0.059**	**0.297**	**0.052**	**0.003**
Mucilaginibacter	0.012	0.009	0.005	0.004	0.019	0.007	0.027
Pedobacter	0.164	0.133	0.051	0.057	0.278	0.047	0.003
**Spirosomaceae**	**0.014**	**0.009**	**0.008**	**0.005**	**0.020**	**0.007**	**0.035**
Lacihabitans	0.014	0.009	0.008	0.005	0.020	0.007	0.035
**Unknown family**	**0.001**	**0.001**	**0.000**	**0.000**	**0.001**	**0.000**	**0.010**
UnculturedST-12K33	0.001	0.001	0.000	0.000	0.001	0.000	0.010
**Weeksellaceae**	**0.022**	**0.020**	**0.009**	**0.013**	**0.036**	**0.016**	**0.046**
Empedobacter	0.022	0.020	0.009	0.013	0.036	0.016	0.046
**Proteobacteria**	**0.761**	**0.168**	**0.744**	**0.082**	**0.779**	**0.252**	**0.420**^**X**^
**Burkholderiaceae**	**0.527**	**0.131**	**0.624**	**0.015**	**0.430**	**0.121**	**0.053**
Alicycliphilus	0.011	0.007	0.006	0.006	0.016	0.003	0.047
Caenimonas	0.067	0.068	0.009	0.008	0.124	0.042	0.019
Simplicispira	0.450	0.186	0.609	0.027	0.291	0.099	0.012
**Rhodobacteraceae**	**0.021**	**0.014**	**0.034**	**0.003**	**0.008**	**0.002**	**0.001**
Amaricoccus	0.008	0.009	0.016	0.007	0.001	0.001	0.034
Thioclava	0.013	0.006	0.018	0.004	0.007	0.002	0.011
**Rickettsiaceae**	**0.001**	**0.001**	**0.000**	**0.000**	**0.002**	**0.001**	**0.049**
Orientia	0.001	0.001	0.000	0.000	0.002	0.001	0.049
**Sphingomonadaceae**	**0.213**	**0.181**	**0.086**	**0.094**	**0.339**	**0.160**	**0.046**
Uncultured	0.213	0.181	0.086	0.094	0.339	0.160	0.046

Mean relative abundance (normalized to the total read percentage per each sample) and its standard error (SEM) are shown for overall samples, I-source, and P-source samples, separately.

* Classification labels for Domain Bacteria (bold = Phylum; suffix -aceae = Family; unbold = genus).

** Average *p* value for each phylum, family, and genus based on one-tail distribution student t-test between I-source and P-source samples (X = non-significant, *p* > 0.05).

The species exhibiting the highest overall relative abundance was *Simplicispira* sp. (0.45), which was up-represented in the I-source, while the uncultured Saprospiraceae bacterium (0.227), *Pedobacter* sp. (0.164), and uncultured Sphingomonadaceae bacterium (0.213) were up-represented in the P-source.

**Figure 4:**
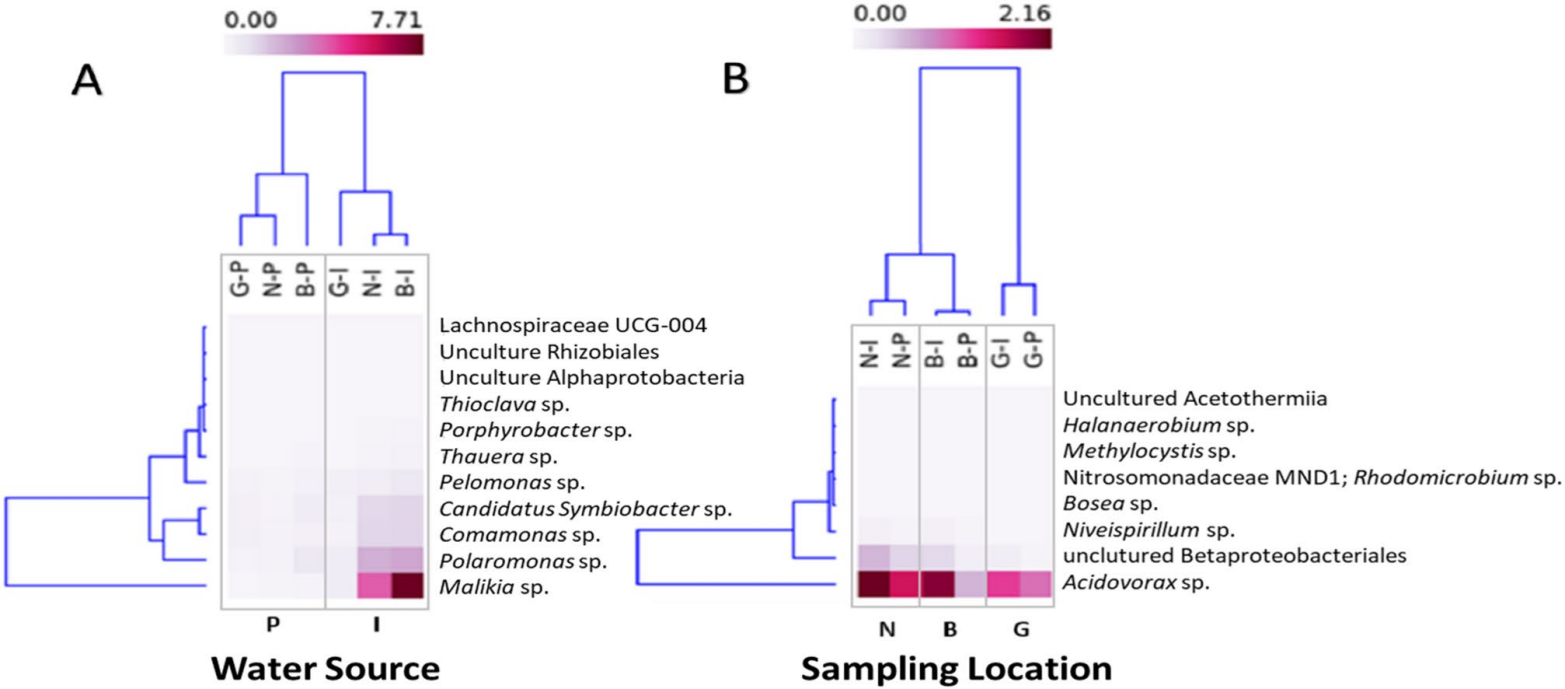
Heatmaps based on Person-multiple correlation analysis among the identified bacterial species. Two heatmaps, one was able to discriminate the I- source samples from the P-source samples and includes 11 species (**A**), and the other discriminates the N, B, and G locations regardless of the water source and includes nine species (**B**).

All data were analyzed using Pearson-based multiple correlation analysis based on the counts of all the identified species and visualized using heatmaps. Samples-based clustering was estimated for several correlation blocks. The single correlation-block that was detected to cluster the samples by location (i.e., N, B, and G) regardless of their source included nine species. Three correlation-blocks were found to cluster the samples by water source (i.e., I or P) regardless of their location, and these included 16 species, one of which included 11 species (Fig. 4). The correlated species were tested for species-species co-occurrence and visualized as a network. One significant connection was formed among five of the 16 species found to distinguish the water source, but none distinguish the sampling location. The detected species belonged to phylum Proteobacteria, *Candidatus Symbiobacter* sp., *Comamonas* sp., and *Polaromonas* sp. (Burkholderiaceae) and *Porphyrobacter* sp. (Sphingomonadaceae), and one species belonged to phylum Firmicutes, Lachnospiraceae bacterium in one connected cluster. The 11 species that did not form a network were Beijerinckiaceae bacterium, Bacteriap25, *Gracilibacter* sp., *Malikia* sp., *Oligoflexus* sp., *Pelomonas* sp., *Polycyclovorans* sp., *Thioclava* sp., *Thauera* sp., uncultured Alpha-proteobacterium, and uncultured Gamma-proteobacterium (JTB255; Fig. 4).

### Influence of samples locations and distance on microbial diversity

The flow of water is northeast; accordingly, the flow of water hypothetically runs first from location N, passes through B, and then finally reaches G. Interestingly, it was observed that both N and B locations shared more OTUs than with the G location (Supplementary Fig. 1). Furthermore, N samples have less OTUs than the B and G sites, which raised a question about the influence of the geographic position and distance on the sampled locations. Based on such a hypothesis, a Euclidean geographic distance matrix was estimated to provide a spatial scale for further correlation analysis. A Mantel test was performed to examine the correlation between the geographic distance between the sampled farms and the number of characteristic species in I- and P-source samples. In the case of the inflow-water samples, no significant (*p* > 0.05) correlation was observed. In contrast, the characteristic species count for pond-water samples significantly correlated with the distance between the samples (r = 0.969, *p* < 0.0001).

## Discussion

The 16S rRNA and ITS metabarcoding analyses were performed to study microbial richness and diversity in pond-waters compared to inflow-water sources from three freshwater semi-intensive aquaculture farms. In general, several bacterial species/genera with major functional and metabolic aspects were identified, including the decomposition of complex organic molecules (Proteobacteria), glycan metabolism (Bacteroidetes), decomposition of carbohydrates (Actinobacteria), polysaccharide-degradation (Verrucomicrobia), and the possible synthesis of cyanotoxin, photosynthesis and oxygen production (Cyanobacteria;^23,24^). Eukaryotic species belonging to three kingdoms were Animalia (amoeba/protozoan), Plantae (algae, diatoms, and higher plants), and Fungi (fish, human, and plant pathogens, and mushrooms/yeasts). Fungi and algae included a group of species that produce volatile compounds. The organisms that characterize the pond-water samples were previously reported to have the ability to produce metabolites, grow in extreme ecological conditions (e.g., Polar regions, deserts, marine water) and/or laboratory contaminants, as well as phyto-, human, and animal pathogens. The relevant species identified from the fishpond-water will be detailed onwards.

Different pollutants present in aquatic environments have a distinct effect on fish microorganisms^25^, causing a reduction in fish immunity against microorganisms^26^. Besides, some pollutants may foster the spread of fish parasites in water^27^. In this context, fish and human infectious eukaryotes were detected in the studied samples. Fish infectious eukaryotes such as *Chilodonella cyprinid* (protozoan parasite), *Metschnikowia bicuspidate* (fungal parasite), *Chrysophyceae* sp. (golden algae parasite), and *Saprolegnia* sp. (water mold) were detected in the current survey.

Pollutants like organic chemicals and heavy metals could be introduced to the aquatic environment from agriculture, domestic and/or industrial activities. The possible presence of pollutants in the aquaculture water investigated here was indicated by species belonging to *Thauera*, *Pelomonas*, and *Malikia* genera and the family Saprospiraceae. Genus *Thauera* includes species that frequently occurred in wet soil and polluted freshwater^28^. Genus *Pelomonas* includes non-spore-forming bacteria from the family Comamonadaceae, which were first isolated from haemodialysis and industrial waters^29^. Furthermore, genus *Malikia* is a rod-shaped motile bacterium isolated from activated sludge of a municipal wastewater treatment plant^30^. Species belonging to the Saprospiraceae family are widely distributed in freshwater and activated sludge^31^. Additionally, human infectious eukaryotes like *Desmodesmus hystrix*, *Malassezia globose*, *Cryptococcus* sp., *Cryptococcus neoformans*, and *Tetrahymena tropicalis* were detected. Pollutants 2-(2-butoxyethoxy)ethanol and 4-bromophenol were previously reported in aquaculture water sampled from the same sources^15^.

In general, the metabolic activity of some detected microbes (e.g., genus *Dysgonomonas*) increases the heavy metal concentrations in aquatic environments and causes water pollution^16,17^. Interesting, counts of species belonging to *Dysgonomonas* genus were significantly higher in the P-source samples, affecting the quality of the related aquaculture products. Furthermore, the high concentration of these metals in freshwater could negatively contribute to fish aroma during storage by promoting lipid oxidation^32^.

Fish malodor is a worldwide problem, and that is routinely observed in several cultivated fish species and is mostly associated with the presence of geosmin (earthy) and 2-methylisoborneol (MIB; musty;^33^). The main causes of these compounds are their production by species belonging to Actinobacteria and Cyanobacteria phyla^8^. Recent reports, however, indicated that other species might also contribute to fish malodor (e.g., members of the genera *Myxobacteria* and *Sorangium* of the phylum Proteobacteria;^21^).

In the present study, all three phyla were detected, namely Actinobacteria, Cyanobacteria, and Proteobacteria, including *Myxobacteria* sp. and *Sorangium* sp. Additionally, the green and golden algae role in producing taste and odor compounds is well documented^34^. For example, *Chloromonas augustae*, detected here, reported to produce odor-active compound by-products (e.g. geosmin and MIB) and serve as an example for the direct influence of some of the eukaryote species on the aroma profile of aquaculture water and its related fish^33^. Besides geosmin and MIB, other compounds with an earthy/musty smell or other off-flavors were recently reported in water, fish, and fish feeds^15–17^. These compounds might be enriched directly as by-products of microbial activities and/or indirectly via oxidation and/or any other chemical reaction for these by-products. In this context, *Sphingomonadaceae* sp. was one of the most highly represented species in P-source samples. Species belonging to this family are generally characterized by their ability to produce sphingolipids^35^. Additionally, oxidation of sphingolipids was reported as a potential off-flavor source^36^. More possible direct contributors to the smell of water were the many mushrooms in the stream, e.g., *Agaricales* fungus, with their mushroom-smelling substances, 1-octen-3-one, and 1-octen-3-ol as key-aroma compounds^37^.

Furthermore, plant infection fungi might have an indirect influence on the aroma of fish and water, where it is well known that the infected plant tissues produce some of the terpenes and other volatile compounds as a defensive mechanism against infection^38^. *Nectria flavoviridis*, the detected wood-decaying fungi, might serve as one example for releasing plant terpenes in the water stream. These results follow a related study in which volatile compounds from plant origins were in aquaculture water sources, including several pyrazines in water gathered from the same sampling points^14^. In the current study, a species belonging to the genus *Capsicum* was detected, and that could be one possible source of these pyrazines, among other sources, as these compounds are known to be produced in high concentrations in peppers^14,15^.

Based on the Mantel test, no significant correlation was observed for the I-source samples. This reflects the homogeneity in the microbial diversity from the river. In contrast, a significant correlation was found between the characteristic species count for pond-water and the distance between the samples. Thus, microbial diversity not only originated from the inflow-water but was also dependent on location-based factors, such as farming practices. Among others, the farming practices in each farm might play a key role in differentiating the samples' biodiversity, e.g., fish-associated microbiota concerning the cultivation of different seasonal fish species and/or using different fish feeds. Unconsumed fish feed reported increasing in nitrogen and phosphorus levels in the water. The elevated levels of these nutrients would enhance microbial growth and, subsequently, severe deoxygenation of the bottom water, thereby stimulating anaerobic microbial activity^2^. This phenomenon could justify the exclusive and the prominent microbial species (both bacteria and fungi) in the P-source samples.

Three of the detected anaerobic bacteria were among the relatively abundant species that were significantly represented in the P-source samples. Two of these, namely *Dysgonomonas* sp. and *Paludibacter* sp., were closely related genera that belong to the phylum Bacteroides^39^. As previously discussed, the *Dysgonomonas* sp. genus has been isolated from human sources^40^, but its presence in water samples was not surprising as other human-related microbes were also detected (e.g., *Empedobacter* sp., Lachnospiraceae bacterium, *Orientia* sp.). *Paludibacter* sp. is a non-motile, strictly anaerobic organism that forms clusters of clones mainly from freshwater sediments and has also been reported in the gut microbiota of the freshwater paddlefish^39^. *Paludibacter* sp. clusters could include any other closely related bacterium (i.e., *Dysgonomonas* sp.), considering that the sediments' levels are higher in fishponds; this might explain the enrichment of both species in pond-water samples^39^. Thus, the significantly high abundance of *Dysgonomonas* sp. in the P-source samples might be due to *Paludibacter* sp. clusters rather than the high presence of a human contaminant.

Meanwhile, species that belong to the genus *Paludibacter*, which is considered primary fermentative bacteria, can degrade dead microbial biomass and produce organic alcohols and fatty acid by-products. These by-products are further metabolized by secondary fermentative bacteria Paludibacteraceae that belongs to the same family. Intermediate products, e.g., propionate and benzoate, ultimately produce acetate and hydrogen^41^. The findings would explain the detection of several fatty-acids oxidation odorants, alcohols, and aldehydes in the inflow-water in a previous study^14^. The third anaerobic bacterium detected was the denitrifying *Alicyclicphilus* sp. This species can utilize aromatic hydrocarbons as carbon sources, which leads to a reduction in organic pollutant levels^42^. In addition to the anaerobically favorable conditions for *Alicyclicphilus* sp., the elevated nitrate and nitrite concentrations in fish ponds might explain its significantly high-representation in P-source samples.

The correlated-block included other related examples as *Gracilibacter* sp. that belong to phylum Firmicutes, an obligatory anaerobic thermotolerant sediment bacterium that receives acid sulfate water^43^. *Oligoflexus* sp. is a nitrous oxide producing species that was isolated from the Sahara Desert^44^. *Beijerinckiaceae* sp. is a free-living aerobic nitrogen-fixing acidotolerant bacterium^45^, while *Thioclava* sp. is a marine anaerobic sulfur-oxidizing bacterium^46^. The *Polycyclovorans* sp. is a marine aromatic hydrocarbon-degrading bacteria^47^, and the uncultured Gammaproteobacterial JTB255 is recorded as a marine benthic bacteria that are responsible for dark carbon fixation in the oceans tidal sediments; both species belong to phylum Proteobacteria^48^.

Based on the present findings, it is hypothesized that fish feed might contribute to microbial diversity in aquaculture water, while this assertion is supported by the results of related studies^34^. In the latter study, the aroma and lipid compositions of the feed used in these three farms were investigated. Whereby it was found that farm N used three types of fish feed, of which two of these feeds were also used in farm G. In contrast, farm B used a single type of feed that was different from those of farms N and G. Unneglected observation was that the only site that exhibited the highest number of species (unique and shared) was farm B. Both feed source and type can explain the microbial profile differences of the P-source samples. Two of these species were microbes of the human intestinal tract (e.g., *Coprococcus* sp. and uncultured *Eubacterium* sp.). Since the inflow-water is clean, the presence of such microbes might be attributed to the individual practices followed in each farm, e.g., type of feed. However, the correlation between these bacteria and the type of feed and relative volatile compounds cannot be currently stated. Further investigations on the microbial composition and related factors of these feeds are needed via the whole-genome shotgun metagenomic sequencing approach (WGS-metagenomics).

In conclusion, the biodiversity and microbial enrichment of eukaryotes and prokaryotes in pond-water are more related to location than the water source. This might be attributed to the individual practices applied at each farm and their surrounding environment. This conclusion might be supported by the exclusive species present in pond sources compared to inflow sources. These species were found to relate to specific characteristics, e.g., lower O_2_ levels. Furthermore, other species allowed the samples to be clustered according to their water type (i.e., I or P) regardless of their location. These species, however, were prominent in the pond source samples and could be associated with pollution and/or pathogenicity for both fish and humans. From an economic point of view, and concerning fish malodor, species from the bacterial domain that might be associated with the production of geosmin and MIB were in all samples but were significantly presented in pond source samples. Besides geosmin and MIB, fatty acid oxidation by-products played a crucial role in fish malodor (i.e., sphingolipid produced by *Sphingomonadaceae* sp.). Plant infected tissues might enrich indirect sources of aroma compounds in water, including terpenes and other volatile compounds, and are considered as a mechanism of defense against infections. It is important to highlight that the role of fungi in malodor formation in aquaculture was not sufficiently studied. These findings draw attention to the complex mechanism of malodor formation and accumulation sources in aquaculture and should be explored in further studies.

## Methods

### Water sampling

**Figure 5:**
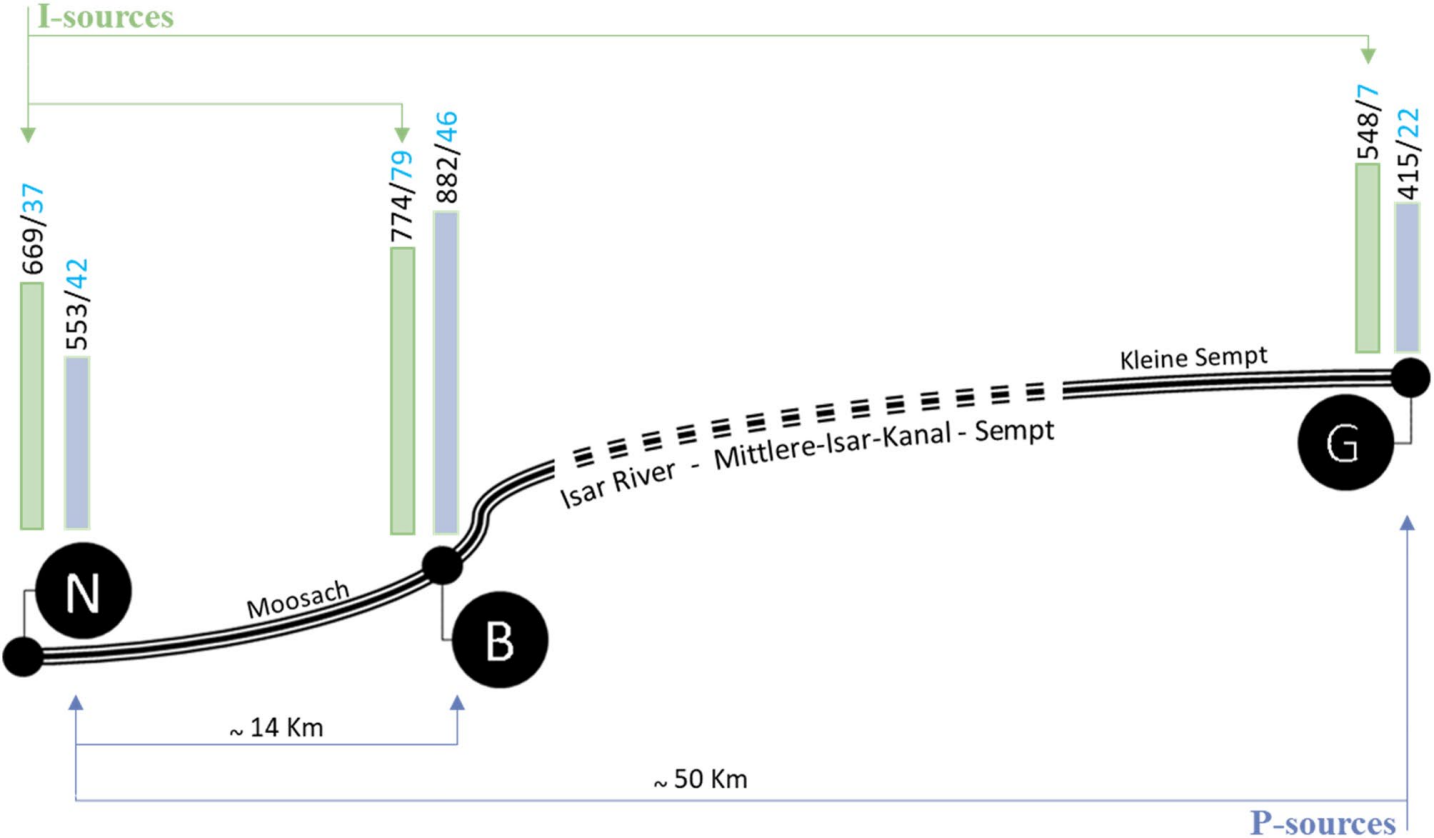
Schematic figure showing the sampling sites and their total number of detected microbial OTUs (bacteria in black/Fungi in blue) in the inflow (I-sources) and ponds water (P-sources) samples.

The focus was to compare pond-water samples (P-source) from three trout farms geographically close to each other, contrasted by inflow-water samples (I-source) gathered from their water sources; the Moosach and Sempt Rivers. Sites N and B are directly located on the Moosach River and are separated by 14 km, whereas the distance between the first site N and the last G is 50 km. Site G is located on the Sempt River and has several connecting small canals with the Moosach, which means that water flow between these small canals occurs, and the flow of water is northeast (Fig. 5). The exact location of these farms cannot be disclosed based on the farmer demand.

Water sampling campaigns were carried out between the end of March and mid-April 2017. Samples were collected at five cm below the water surface using individual 500 ml sterile disposable plastic bottles. Triplicate samples were collected from the three inflow-water sources (labeled N-I, B-I, and G-I) and three from pond-waters (labeled N-P, B-P, and G-P). In the inflow-water, samples were gathered at the pond edge near the inflow-water entrance where the feed is usually offered. The triplicate samples from each site were bulked and homogenized in equal portions for NGS analysis. We followed the same methodology of sample preparation for metagenomic analysis, as previously reported^37^.

### DNA extraction, library preparation, sequencing, and quality control

Samples were packed and sent to NGS services (Macrogen Inc., South Korea) for DNA extraction and the 16S rRNA and ITS metabarcoding analyses. The DNA was extracted from the inflow- and pond-water samples using a DNA isolation kit PowerSoil (MOBIO, No. 12888-100, USA) according to the manufacturer manual. The extracts that passed the quality control (QC) were processed for library construction. The PCR amplicons were prepared by amplifying the V3-V4 region of the 16S prokaryotic rDNA gene and ITS2 spacer of the 45S eukaryotic rDNA gene using KAPA HiFi Hot Start Ready Mix PCR Mix kit (16S primers: Bakt_341F (5′-CCT ACG GGN GGC WGC AG-3′) and Bakt_805R (5′-GAC TAC HVG GGT ATC TAA TCC-3′); ITS2 primers: ITS3F (5′-GCA TCG ATG AAG AAC GCA GC-3′) and ITS4R (5′-TCC TCC GCT TAT TGA TAT GC-3′). The library preparation and optimization were performed using the Nextera XT DNA Library Preparation Kit and sequenced on MiSeq 3000 (Illumina, Inc., USA).

MiSeq Control Software v2.2 (Illumina, Inc., USA) was used to generate raw images for system control and base calling and converted into fastq format using bcl2fastq v1.8.4 (Illumina, Inc., USA). Scythe (v0.994; available at http://github.com/vsbuffalo/scythe) and Sickle (available at https://github.com/najoshi/sickle) software were used to remove the adapter sequences, while sequences below 36 bp were discarded. The sequencing quality score of a given base, Q, is defined by the estimated probability of the base call being wrong. Higher Q scores indicate a smaller probability of error. The sequencing output and quality stats for each sample are presented in the supplementary table 1. The pair-end fastq files were paired, merged using BBmerge tool at a normal merge rate, and trimmed for low quality (Q 30) while discarding short reads (below 50 bp) using BBduk tools in Geneious Prime to generate compressed, clean, merged, and filtered fastq files^49^.

### Bioinformatics pipeline

For prokaryote analysis (16S), SILVAngs was used for data analysis using the compressed fastq files through an online automatic software pipeline based on the SILVA rDNA database at default settings^50^. Each read was aligned against the SILVA SSU rRNA using the SILVA Incremental Aligner (SINA v1.2.10;^51^). Short reads and/or with more than 2% ambiguities or homopolymers were discarded. Followed by a dereplication for the identical reads, then the unique reads were clustered and assigned as OTUs (organism taxonomical units) using cd-hit-est v3.1.2;^52^) running in the accurate mode, ignoring overhangs and applying identity criteria of 1.00 and 0.98, respectively. The reference read for each OTU was classified for each sample based on local BLASTn search against the non-redundant version of SILVA SSU ref dataset (release no. 132) with standard-setting^53^. All reads were subject to mapping using the OTU reference reads to yield the quantitative information for each OTU per sample using BLASTn features. Samples with no or weak BLAST hits where the function "(% sequence identity + % alignment coverage)/2" did not exceed the value of 93 remained unclassified or unassigned.

For eukaryotes (ITS), a customized pipeline based on Geneious Prime Platform was designed and applied as follows. The filtered fastq were dereplicated using Dedupe tool (kmer set length at 31) from BBmap package^54^. The unique reads were de novo assembled using SPAdes default parameters for metagenomic data^55^. Generated consensus sequences were subject to the "Classify Sequences" tool using the Warcup V2 training data set with medium sensitivity (find matches with 90% similarity) and a minimum overlap of 75 bp.

### Data treatment and statistical analysis

Alpha-diversity is used to identify the species diversity in sites or habitats at a local scale and was calculated according to the Simpson diversity index^56^. Beta-diversity (true beta-diversity) is the ratio between regional and local species diversity and is calculated according to the Bray Curtis measures^57^. Both diversity indices are based on the relative abundances and the different species identified; thus, they were applied only to the prokaryotes detected in the current study. For eukaryotes, as each species' counts are technically not possible due to the multi-cellular nature of the eukaryotic organism, only species richness was determined for each sample.

In prokaryotes, after normalizing the counts to the total reads per sample, two methods were used to detect water-source related microbial species: (a) *Refining and applying t-test*: Venn diagrams were used to define the unique and shared species among locations and/or water sources. Then, the shared species were used for t-test among both sources; (b) *Multiple-correlation-based distance:* Heatmap blocks reflecting a high degree of correlations were tested for their ability to discriminate the water source and/or sampling locations; correlations-blocks of interest were visualized as separate heatmaps and were subject to network plotting.

In eukaryotes, shared and unique species IDs were defined using Venn diagrams. Generally, Venn diagrams and t-test were performed using the Microsoft Excel program, while the heat-maps, Pearson-based correlation, and network analysis were performed using Orange software^58^. The isolation by distance (IBD) was applied to check the influence of the geographic distance and/or the water source type on the beta-diversity among the three sampled locations. The correlation between the Euclidean distances of the I- and P- samples and the geographic distance between the three locations were tested using a Mantel test and performed using IBD v1.52^59^ and XLSTAT 2017 (Addinsoft, Paris, France) software.

## Supplementary Information

Supplementary Information.

## Data Availability

Data is available at NCBI SRA database, BioProject: PRJNA589169. https://www.ncbi.nlm.nih.gov/Traces/study/?acc=PRJNA589169&o=acc_s%3Aa.
