# Supplementary material for: Metabarcoding profiling of microbial diversity associated with trout fish farming
Source: Sci Rep. 2021 Jan 11;11:421. doi: 10.1038/s41598-020-80236-x (PMC7801479; doi:10.1038/s41598-020-80236-x)
Supplement: Supplementary file 1 — Supplementary Information. [file 41598_2020_80236_MOESM1_ESM.docx]

**Supplementary File**

**Article Title:** Metabarcoding Profiling of Microbial Diversity Associated With Trout Fish Farming

**Authors:** Mohamed A. A. Mahmoud, Mahmoud Magdy


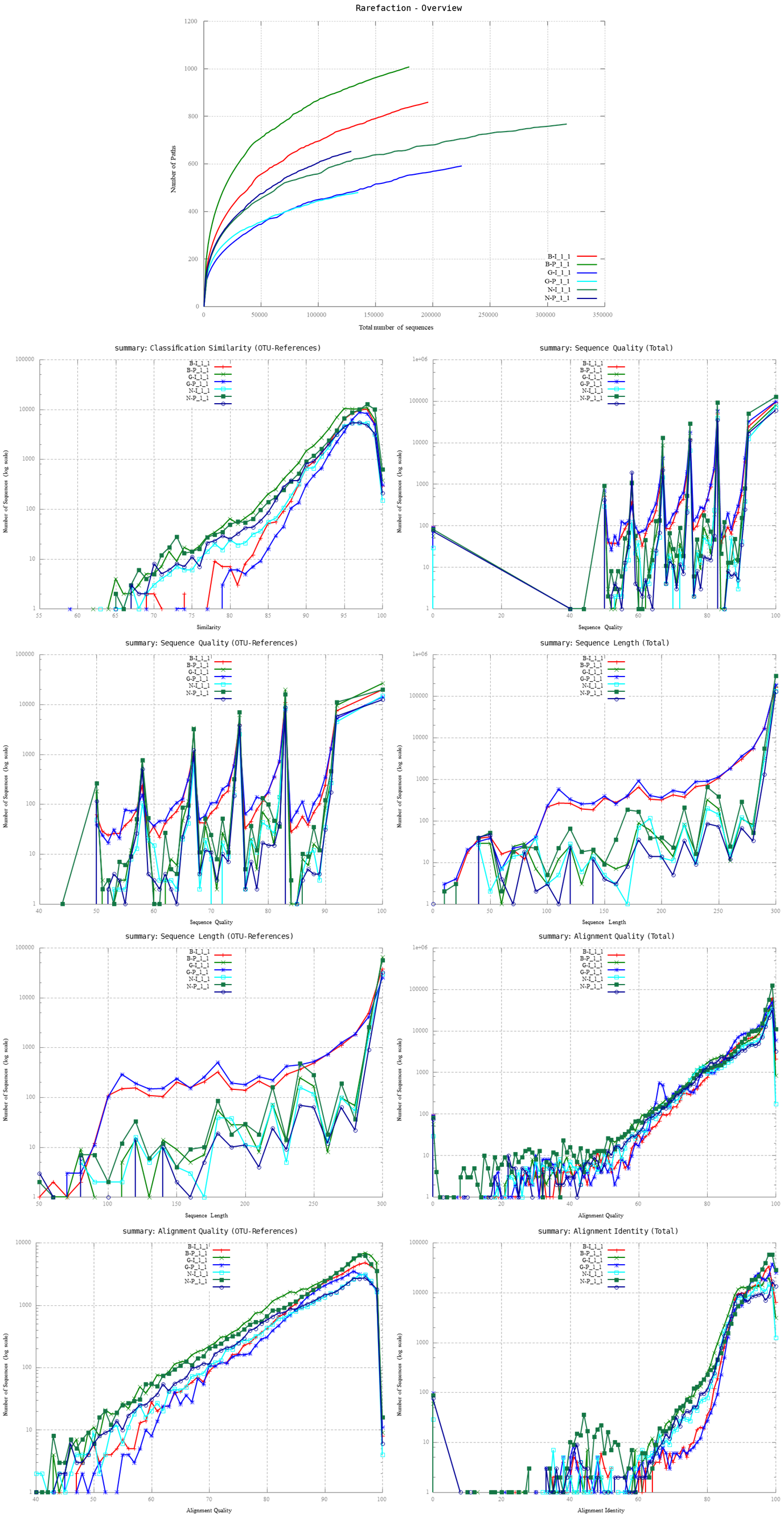


**Supplementary Figure 1.** SILVAngs pipeline quality control output graphs per sample, include the rarefaction number and the classification summary, in addition to sequence length, alignment quality and alignment identity based on OUT-references and the total sequences, respectively.

**Supplementary Table 1.** Metabarcoding data statistics. The sampling site, amplicon target (16S for prokaryotes or ITS for eukaryotes), the water sampling source, total number of bases in million base pairs, total number of reads and reads after adapter trimming, GC (%) after adapter trimming, ratio of reads that have phred quality score of over Q20 (%) after adapter trimming, ratio of reads that have phred quality score of over Q30 (%) after adapter trimming, and the SRA accession number are shown.

| Sampling site | Amplicon target | Water source | Total bases (Mbp) | Total reads | Trimmed reads | GC (%) | AT (%) | Q20 (%) | Q30 (%) | SRA accession number |
| --- | --- | --- | --- | --- | --- | --- | --- | --- | --- | --- |
| G | 16S | Inflow | 138 | 458,654 | 451,498 | 52.524 | 47.48 | 91.036 | 83.696 | SRR10447746 |
|  | ITS | Inflow | 114 | 381,362 | 360,626 | 45.698 | 54.3 | 93.031 | 86.974 | SRR10447754 |
|  | 16S | Fishpond | 82 | 273,594 | 269,972 | 52.087 | 47.91 | 92.586 | 85.625 | SRR10447745 |
|  | ITS | Fishpond | 112 | 374,284 | 357,184 | 46.595 | 53.41 | 93.208 | 87.261 | SRR10447753 |
| N | 16S | Inflow | 193 | 642,590 | 635,396 | 53.744 | 46.26 | 91.118 | 83.655 | SRR10447756 |
|  | ITS | Inflow | 126 | 420,940 | 380,144 | 46.062 | 53.94 | 91.631 | 85.553 | SRR10447752 |
|  | 16S | Fishpond | 81 | 269,112 | 257,682 | 51.757 | 48.24 | 91.699 | 84.425 | SRR10447755 |
|  | ITS | Fishpond | 116 | 388,636 | 355,234 | 42.945 | 57.05 | 92.244 | 86.222 | SRR10447751 |
| B | 16S | Inflow | 119 | 398,274 | 392,524 | 53.536 | 46.46 | 91.434 | 84.171 | SRR10447750 |
|  | ITS | Inflow | 127 | 424,348 | 402,356 | 48.873 | 51.13 | 91.408 | 84.965 | SRR10447748 |
|  | 16S | Fishpond | 109 | 363,946 | 359,408 | 53.066 | 46.93 | 91.442 | 83.928 | SRR10447749 |
|  | ITS | Fishpond | 110 | 368,054 | 339,716 | 46.123 | 53.88 | 92.915 | 86.775 | SRR10447747 |
